# Vancomycin- and piperacillin-induced acute interstitial nephritis in a patient with lupus: A case report showcasing rapid decline in renal function 

**DOI:** 10.5414/CNCS111180

**Published:** 2023-06-08

**Authors:** Oluwadamilola Adisa, Anil Ananthaneni, Bryce Rushing, Nathan Rinehouse, Phani Morisetti

**Affiliations:** 1 Resident, Department of Internal Medicine, Louisiana State University and Health Sciences Center, Shreveport, LA,; 2 Resident, Department of Internal Medicine, Emory University School of Medicine, Atlanta, GA,; 3 Resident, Department of Pathology, and; 4 Nephrologist, Division of Nephrology and Hypertension, Louisiana State University and Health Sciences Center, Shreveport, LA, USA

**Keywords:** vancomycin, piperacillin, AKI, acute interstitial nephritis

## Abstract

Drug-induced acute interstitial nephritis (AIN) presents as acute kidney injury (AKI) with the use of certain offending drugs. Antibiotics, such as β-lactams, trimethoprim-sulfamethoxazole, fluoroquinolones, and rifampin, account for up to 50% of drug-induced AIN cases. The onset of drug-induced AIN following drug exposure usually ranges from few days to several weeks or months. We present a patient with lupus who had rapid decline in renal function with a single dose of vancomycin and piperacillin-tazobactam (VPT) administration, termed as the “workhorse” regimen at many institutions. In addition, she did not exhibit many clinical and laboratory signs of AIN, making diagnosis challenging. Prompt kidney biopsy and early steroid therapy had a critical role in recovery of the patient’s renal function. The median duration for renal impairment in vancomycin-induced AIN is 26 days. Onset of AKI is usually rapid from VPT, within 3 – 5 days of drug exposure. However, the severity of AKI is often low, in contrast to this patient whose AKI reached a stage 3 (AKIN/KDIGO) within 2 days from drug exposure. This study highlights the nephrotoxic potential of piperacillin, especially when used along with vancomycin, concurrent with recent evidence. Within rising antibiotic usage rates, is important to consider AIN in the differential diagnosis of rapidly declining AKI, especially with the combined use of VPT.

## Introduction 

Drug-induced acute interstitial nephritis (AIN) presents as acute kidney injury (AKI) with the use of certain offending drugs and may be associated with the classic triad of fever, rash, and eosinophilia [1]. Systemic signs, such as maculopapular rash, eosinophilia, and eosinophiluria, are present only in less than one-third of cases [2, 3]. The most common antibiotic drugs implicated are penicillins, cephalosporins, rifampin, sulfonamides, and ciprofloxacin. The development of AIN is not dose-dependent and can recur with a second exposure to the same or related drug [4]. The onset of drug-induced AIN following drug exposure may range from 3 to 5 days (as occurs with a second exposure to an offending drug), to as long as several weeks, to many months (as occurs following a first exposure to an offending drug) [5]. In our literature review, there have been very few reports of vancomycin- and piperacillin-associated AIN with precipitous decline in renal function needing dialysis. 

## Case presentation 

A 41-year-old female with a history of discoid lupus presented with progressively worsening left knee pain and swelling for 2 days. She reported subjective fevers, chills, and myalgia. She denied recent falls/trauma, exposure to sick contacts, or previous similar episodes. On presentation, she was febrile at 38.6 °C (101.5 °F) and tachycardic at 136 bpm. Her left knee was swollen, warm to touch with restricted range of motion. Complete blood count (CBC) showed hemoglobin (Hgb) 12.3 g/dL, platelet (PLT) 209 K/µL, white blood cell (WBC) 5.79 K/µL with differentials count; granulocytes 4.1 K/µL, lymphocytes 1.1 K/µL and monocytes 0.6 K/µL. Basic metabolic panel (BMP) was grossly unremarkable with liver function tests (LFTs) showing aspartate amino transferase (AST) 29 U/L, alanine aminotransferase (ALT) 51 U/L, and alkaline phosphatase (ALP) 91 U/L. Procalcitonin was unremarkable. Urinalysis was not suggestive of infection. erythrocyte sedimentation rate (ESR) and C-reactive protein (CRP) were elevated at 65 mm/h and 16.9 md/dL, respectively. In the emergency department, the patient received a single dose of vancomycin and piperacillin-tazobactam (VPT) empirically. Left knee X-ray showed moderate suprapatellar joint effusion and soft tissue swelling. Aspirate of synovial fluid was bloody, and cytology analysis revealed 1,400/mm^3^ with 57% segmented cells and no crystals, essentially ruling out an infectious etiology and gout. Blood and synovial cultures were unremarkable. 

Upon admission to General Medicine, antibiotics were stopped, and rheumatology was consulted. ANA, anti-SSA, and RNP-antibodies were positive. Complement levels, anti-dsANA, and vasculitis panel were unremarkable. She had symptomatic improvement of knee complaints following aspiration of effusion and pain management. However, on day 2 of admission, serum creatinine (Cr) was noted to have increased from 0.87 to 1.4 mg/dL and continued to rise to 5.4 and 8.5 mg/dL on day 3 and 4, respectively. Given elevated inflammatory markers and suspicion for lupus nephritis/AIN, rheumatology service had recommended a trial of steroids started on day 3. Renal ultrasound showed enlarged kidneys with slightly increased parenchymal echogenicity with poor corticomedullary demarcation (nonspecific finding but associated with interstitial infiltration, interstitial fibrosis, or tubular atrophy). 

**Figure 1.**
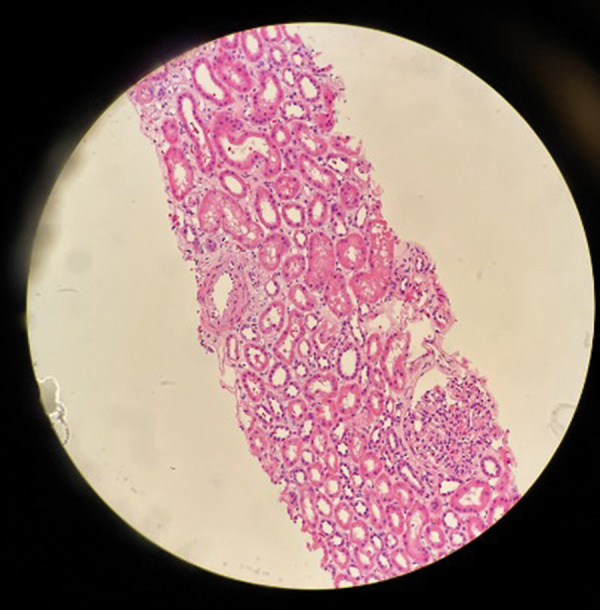
H & E photomicrograph showing mild edema and focal tubular injury, evidenced by cytoplasmic vacuolization, slightly dilated tubules with a thin epithelial lining, and diffuse loss of the brush border. The glomeruli are normal with wide-open loops and smooth, thin capillary membranes.

**Figure 2.**
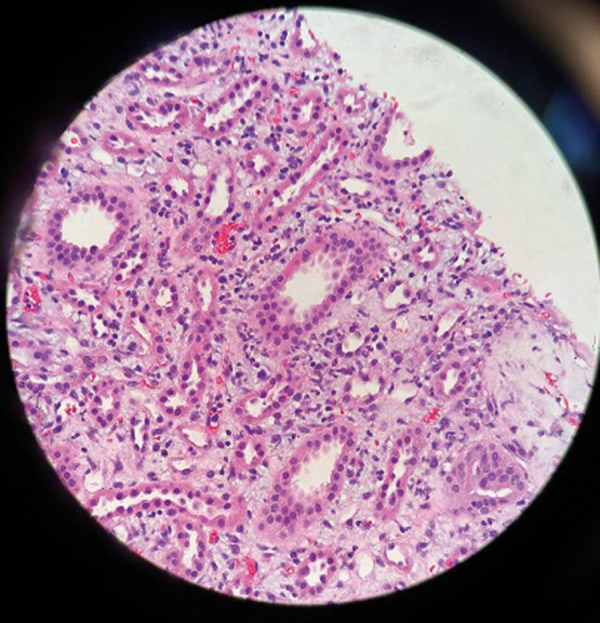
H & E photomicrograph showing mild edema and focal inflammatory infiltrate. Several neutrophils are identifiable.

**Figure 3.**
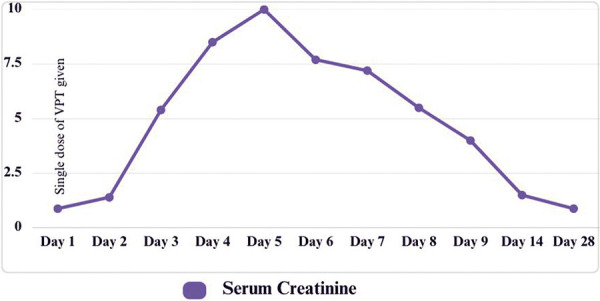
Patient’s serum creatinine trend during hospitalization and during follow-up visits after discharge.

With failed improvement and rapidly declining renal function despite steroids, nephrology service was consulted. Urinalysis showed 1+ protein, 3+ blood, RBC 24/HPF, and urine protein/creatinine ratio 0.59. Urine sediment showed muddy brown casts and non-dysmorphic RBCs. She started to have decreasing urine output, and AKI was unresponsive to hydration and steroids. On day 5, Cr peaked at 10 mg/dL, necessitating one session of hemodialysis during her hospital stay. Renal biopsy performed on day 6 revealed focal edema of the interstitium with dense inflammatory infiltration. Inflammatory cells were predominately lymphocytes. Rare eosinophils and focal tubular injury were noted, with no significant interstitial fibrosis, which confirmed the diagnosis of AIN (Figures 1, Figure 2). With continued steroid use, her Cr gradually trended down to 4 mg/dL by day 9. She was discharged on a steroid course. During a subsequent follow-up visit with nephrology, her Cr had improved to 1.5 mg/dL within 1 week of discharge. Upon her visit to the rheumatology clinic 2 weeks later, it was observed that her serum creatinine had reverted to its baseline level of 0.88 mg/dL (Figure 3). 

## Discussion 

AIN is a form of AKI in which there is an inflammatory infiltrate within the kidney interstitium. The classic triad of fever, rash, and eosinophilia is only present in ~ 10% of cases, but each component alone appears in roughly 25 – 35% of cases [1]. Our patient did not demonstrate any of these presenting features, although she was febrile on arrival and had been treated with acetaminophen during her entire stay, which may have masked AIN-induced fever. Generalized arthralgias are another presenting feature in AIN, occurring in roughly 45% of patients. Laboratory tests are much more useful in diagnosing AIN than clinical presentation alone. Virtually all patients exhibit a rise in creatinine and have a characteristic urine sediment of white cells, red cells, and white cell casts. Our patient was unique in that she did not have white cells or white cell casts on several serial urinalysis. Moreover, patients exhibit a variable degree of proteinuria, from none to > 1 g/day; our patient had 3+ proteinuria, which is significant [6]. 

Antibiotics, such as β-lactams, trimethoprim-sulfamethoxazole, fluoroquinolones, and rifampin, account for up to 50% of drug-induced AIN cases. Other common drugs that cause AIN include loop diuretics, thiazide diuretics, proton pump inhibitors, cimetidine, allopurinol, 5-aminosalicylates, and immune checkpoint inhibitors [7]. More importantly, the development of drug-induced AIN is not dose-dependent, so even a single dose of the above medications can cause AIN [4], as is evident with our patient. 

Vancomycin itself is not classically associated with AIN; rather, it has been long known to cause acute tubular necrosis (ATN) and cast nephropathy when administered concomitantly with other antibiotics. Luque et al. [8] demonstrated through both mouse models and human kidney biopsy samples from patients with severe AKI that vancomycin produces obstructive intraluminal casts with uromodulin. In addition to this tubular damage, interstitial inflammation was also evident, suggesting a hybrid of ATN and AIN (necroinflammation) as the mechanism in our patient. Furthermore, only a few case reports on vancomycin-induced AIN have ever been published, but the median duration of treatment prior to AIN was 26 days, far greater than that of our patient [9]. 

As stated previously, vancomycin’s nephrotoxicity is potentiated with concomitant use of other antibiotics; the most-used therapeutic combination is VPT [10]. Only recently has this combination started being reported as a cause of AKI, occurring in 22.2% of patients treated with VPT compared to 12.9% for other vancomycin combinations. Blair et al. [11] demonstrated through various meta-analyses that VPT can cause ATN, AIN, or a mixed pathology. Onset of AKI is generally more rapid — within 3 – 5 days of commencement of therapy — than with vancomycin alone, as seen in our patient. There is also strong evidence that supports a synergistic mechanism for nephrotoxicity with VPT, but the severity of AKI is often low, which was unfortunately not true for our patient, whose AKI reached a stage 3 (AKIN/KDIGO) within 2 days of drug exposure. 

Once a diagnosis of AIN is established, treatment must be initiated to prevent any further renal damage. Identification and withdrawal of the offending agent are the cornerstone for initial management in the case of drug-induced AIN. Early initiation of corticosteroid therapy, preferably within the first 7 days of initial insult, is the second-most important step in treatment of AIN, as a delay in initiation often leads to worse recovery of kidney function. These patients must also undergo renal biopsy to rule out other causes of renal failure, as did our patient, whose results were wholly consistent with AIN. Recent studies have recommended 0.5 – 1.0 mg/kg/day of methylprednisolone for 3 weeks followed by a 2 – 6-week taper. Pulse dosing of an additional 250 – 500 mg/day of methylprednisolone for 2 – 4 days preceding maintenance therapy has also been shown to improve kidney function by 1 week, but by weeks 2 and 3 the degree of recovery from baseline is like that of patients not treated with pulse dosing. Additionally, maintenance of steroid therapy for longer than 3 weeks or a tapering period beyond 5 – 6 weeks does not confer greater kidney recovery [6, 12]. Patients must then be monitored closely for response to therapy through daily renal function panels. If the patient fails to respond, then another etiology of AKI must be considered and investigated through biopsy. If AIN is still the culprit, then alternative therapies include other immunosuppressants, though very little data exist to support use of this modality. 

Finally, it should go without saying that antimicrobial stewardship should be always observed, as broad empiric coverage is not always indicated. Other measures to minimize the risk of AIN include monitoring daily creatinine levels, pharmacist review, and substitution for less nephrotoxic agents, all of which have been shown to reduce incidence of drug-associated AKI by 23.8% [13]. Additionally, AUC-guided dosing, rather than trough-guided dosing, of vancomycin has recently been shown to reduce nephrotoxicity without compromising efficacy, though laborious and costly [14]. 

## Conclusion 

This study highlights the importance of recognizing the nephrotoxic potential of piperacillin-tazobactam especially when used along with vancomycin, concurrent with recent evidence. Given the increasing use of antibiotics, it is important to consider AIN as a potential etiology for precipitous decline in renal function, even in the absence of many known clinical and laboratory signs of AIN. Withdrawal of any suspicious agent is imperative as a component of conservative therapy. A renal biopsy is warranted in cases where conservative treatment fails to yield improvement within a span of 5 – 7 days. In biopsy-proven cases, steroid therapy is begun and usually continued for 4 – 6 weeks and tapered over the next 4 weeks. Our patient had rapidly declining renal function after a single dose of VPT. She recuperated gradually and significantly after 1 session of hemodialysis and continuation of steroid therapy. 

## Ethical disclosure 

Informed consent was obtained verbally from the patient directly. In addition, our Institutional Review Board does not need consent for sufficiently anonymized case studies. 

## Funding 

This case study received no specific grant from any funding agency in the public, commercial, or not-for-profit sectors. 

## Conflict of interest 

On behalf of all authors, the corresponding author states that there is no conflict of interest.
